# Circulating glutamate concentration as a biomarker of visceral obesity and associated metabolic alterations

**DOI:** 10.1186/s12986-018-0316-5

**Published:** 2018-11-06

**Authors:** Ina Maltais-Payette, Marie-Michèle Boulet, Cornelia Prehn, Jerzy Adamski, André Tchernof

**Affiliations:** 1 Quebec Heart and Lung Institute, Laval University, 2725 Chemin Sainte-Foy, Québec, QC G1V 4G5 Canada; 2 School of Nutrition, Laval University, Québec, Canada; 3 INSA, University Claude Bernard Lyon 1, Villeurbanne, France; 4 Helmholtz Zentrum München, Institute of Experimental Genetics, Genome Analysis Center, Neuherberg, Oberschleißheim, Germany

**Keywords:** Glutamate, Metabolomics, Branched-chain amino acids, Visceral obesity, Waist circumference

## Abstract

**Background:**

Visceral adipose tissue (VAT) area is a strong predictor of obesity-related cardiometabolic alterations, but its measurement is costly, time consuming and, in some cases, involves radiation exposure. Glutamate, a by-product of branched-chain-amino-acid (BCAA) catabolism, has been shown to be increased in visceral obese individuals. In this follow-up data analysis, we aimed to investigate the ability of plasma glutamate to identify individuals with visceral obesity and concomitant metabolic alterations.

**Methods:**

Measurements of adiposity, targeted blood metabolomics and cardiometabolic risk factors were performed in 59 healthy middle-aged women. Visceral and subcutaneous adipose tissue areas were measured by computed tomography (CT) whereas body fat and lean mass were assessed by dual-energy x-ray absorptiometry (DEXA).

**Results:**

The univariate Pearson correlation coefficient between glutamate and VAT area was *r* = 0.46 (*p* < 0.001) and it was *r* = 0.36 (*p* = 0.006) when adjusted for total body fat mass. Glutamate allowed to identify individuals with VAT areas ≥100 cm^2^ (ROC_AUC: 0.78, 95% CI: 0.66–0.91) and VAT ≥130 cm^2^ (ROC_AUC: 0.71, 95% CI: 0.56–0.87). The optimal glutamate concentration threshold determined from the ROC curve (glutamate ≥34.6 μmol/L) had a greater sensitivity than the metabolic syndrome (MetS) and the hypertriglyceridemic waist (HTW) phenotype to identify individuals with VAT ≥100 cm^2^ (83% for glutamate vs 52% for the MetS and 35% for the HTW). Variance analysis showed that women with a high circulating glutamate level (≥34.6 μmol/L) had an altered metabolic profile, particularly regarding total triglyceride levels and the amount of triglycerides and cholesterol in very-low-density lipoproteins (all *p* < 0.01).

**Conclusion:**

Circulating glutamate is strongly associated with VAT area and may represent a potential screening tool for visceral obesity and alterations of the metabolic profile.

## Background

Obesity is associated with an increased cardiometabolic risk [1, 2]. This association is, however, heterogeneous and it is now increasingly recognized that accumulation of abdominal fat and more precisely visceral adipose tissue (VAT) is a very strong indicator of metabolic dysfunction [3]. Precise assessment of VAT accumulation by imaging methods is not feasible on a large scale because it is costly, time consuming and, in some cases, involves radiation exposure [4]. Therefore, simple and accurate VAT predicting tools are still needed.

Studies focusing on metabolomics and the metabolic alterations linked to obesity or body fat distribution have found that obese individuals are characterized by higher circulating levels of branched-chained amino acids (BCAA; valine, leucine and isoleucine) and related metabolites [5]. Moreover, BCAA levels have been shown to decrease upon weight loss by either bariatric surgery [6] or diet [7]. Interestingly, glutamate, a by-product of BCAA catabolism, has been shown to be increased especially in visceral obesity [8–10].

In a large study assessing VAT with computed tomography (CT) in 1449 Japanese subjects, Yakamado et al. found that glutamate was the single metabolite most strongly correlated with VAT area (*r* = 0.49, *p* < 0.001) [8]. They also reported that an amino acid index (AAindex) combining glutamate with 7 other amino acids (valine, leucine, isoleucine, glycine, alanine, tyrosine and tryptophan) could help identify individuals with excessive VAT accumulation. Takashina et al. reported similar results on 83 Japanese adults with normal glucose tolerance. Indeed, glutamate showed the strongest association with VAT volume (measured by magnetic resonance imaging, MRI) among all the metabolites tested (*r* = 0.568, *p* < 0.001) [9]. We reported consistent results in the present sample of 59 healthy Caucasian women for which glutamate concentrations once again was the strongest correlate of CT-measured VAT area (*r* = 0.46, *p* < 0.001) [10]. Considering these independent and consistent results, we investigated the potential of circulating glutamate concentration as a screening tool for excessive VAT accumulation and concomitant metabolic alterations. In a follow-up analysis of our sample [10], we tested the hypothesis that glutamate is strongly and independently associated with VAT and accurately identifies patients with visceral obesity and an altered metabolic profile.

## Methods

This is a new analysis of a dataset for which the recruitment and metabolic assessment details have already been described elsewhere [10]. Briefly, adiposity, targeted blood metabolomics and cardiometabolic risk factors of 59 healthy women undergoing gynaecological surgery were assessed. Total body fat and lean mass were measured by dual-energy x-ray absorptiometry (DEXA). VAT and subcutaneous adipose tissue (SAT) areas were determined by CT at the L_4_L_5_ vertebrae level. Plasma lipid and lipoprotein levels were obtained from 12 h fasting blood samples as previously described [10]. Amino acid levels were determined by targeted metabolomics using the Absolute IDQ kit p180 (Biocrates, Innsbruck, Austria) as described [10]. Alcohol consumption frequency (occasional, frequent or regular) and smoking status (yes or no) were assessed by questionnaire. Menopausal status (pre-, peri- or post-menopausal) was determined by measurement of the follicle-stimulating hormone (FSH) level and the reported presence/absence of menstrual bleeding. Menopausal status was missing for 2 women. The AAindex was calculated as (− 3.5250) + (0.0379*glutamate) + (− 0.0070*glycine) + (0.0034*alanine) + (0.0196*tyrosine) + (− 0.0216*tryptophan) + (0.0054*BCAA) [8]. The cardiometabolic risk stratification algorithms used were the metabolic syndrome (MetS) and the hypertriglyceridemic waist (HTW) phenotype. Presence of the MetS was established with the NCEP-ATP III criteria [11], i.e. 3 or more of the following features: waist circumference (WC) > 88 cm, triglycerides (TG) ≥1.7 mmol/L, high-density lipoprotein (HDL) < 1.3 mmol/L, fasting glucose ≥5.6 mmol/L and diastolic blood pressure (DBP) ≥130 mmHg or systolic blood pressure (SBP) ≥85 mmHg. Presence of the HTW phenotype was defined according to values proposed by Blackburn et al. in 2008 [12]; WC ≥85 cm and TG ≥1.5 mmol/L, as they were obtained in a study sample similar to ours. VAT area thresholds tested were ≥100 cm^2^ and ≥130 cm^2^ because they have been associated with increased cardiometabolic risk [13].

Pearson’s correlation coefficient was used to assess the association of glutamate concentration with VAT. Logistic regression analyses were used to determine the receiving operator characteristic (ROC) curves of glutamate concentration ability to identify individuals with excessive VAT accumulation. The optimal glutamate threshold was determined using Youden’s Index (J), which measures the distance between the cut-off points and the line of equality (diagonal line) and is calculated as J = sensitivity + specificity – 1. The value with the highest J being the cut-off point with the best differentiation ability when equal weight is given to sensitivity and specificity [14]. Sensitivity and specificity were defined as true positive/(true positive + false negative) and true negative/(true negative + false positive) respectively. Women were classified as having high or low glutamate level according to the optimal threshold and analyses of variance (ANOVA) were used to compare adiposity and lipid profiles between groups. Variables were transformed using Log10 or BoxCox to obtain normal distribution when needed. Data are presented as mean ± standard deviation when they were normally distributed and median (min-max) when they were not. Test results were considered significant when *p*-value was ≤0.05. All statistical analyses were performed using JMP software (SAS Institute, Cary, NC).

## Results

Participant characteristics have already been described in detail elsewhere [10]. In brief, all participants were female, mean age was 47.0 ± 5.0 years, median BMI was 26.4 (20.2–41.1) kg/m^2^, median VAT was 89.3 (33.6–278.1) cm^2^, median glutamate level was 35.0 (9.4–93.7) μmol/L, mean fasting glucose was 5.5 ± 0.58 mmol/L, median fasting insulinemia was 7.09 (4.71–10.74) μU/mL and median HOMA-IR was 1.72 (1.12–2.55). Thirty nine women were pre- or perimenopausal and 17 were postmenopausal. The univariate Pearson correlation coefficient between circulating glutamate and VAT area was *r* = 0.46 (*p* < 0.001) and it was *r* = 0.36 (*p* = 0.006) when adjusted for body fat mass. Interestingly, glutamate was only moderately correlated with SAT area (*r* = 0.33, *p* = 0.013) and adjustment for fat mass rendered the correlation not significant (*r* = − 0.01, *p* = 0.937). Glutamate level did not differ according to smoking status (*p* = 0.333), alcohol consumption frequency (*p* = 0.727) or menopausal status (*p* = 0.112). Glutamate was not significantly correlated to SBP (*r* = 0.12, *p* = 0.369) or DBP (*r* = 0.21, *p* = 0.106).

### Logistic regression analysis

**Fig. 1:**
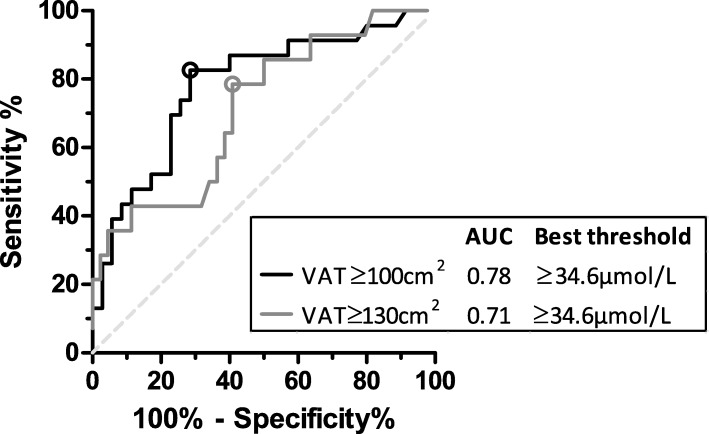
ROC curves of the ability of glutamate concentrations to identify individuals with excessive VAT accumulation. Best thresholds are represented by circles and were determined using Youden’s Index (J = sensitivity + specificity – 1). Sensitivity and specificity were defined as true positive/(true positive + false negative) and true negative/(true negative + false positive) respectively. *ROC* receiving operator characteristic, *VAT* visceral adipose tissue, *AUC* area under the curve

Figure 1 shows ROC curves of glutamate as a continuous variable with the two VAT area thresholds (100 cm^2^ and 130 cm^2^). The area under the curve (ROC_AUC) was 0.78 (95% CI: 0.66–0.91) for VAT ≥100 cm^2^ and 0.71 (95% CI: 0.56–0.87) for VAT ≥130 cm^2^. The best glutamate threshold, defined as the ROC curve’s point with the highest Youden Index, was glutamate concentration ≥ 34.6 μmol/L for both VAT area thresholds. This cut-off point was very close to the glutamate level median (35.0 μmol/L) in this sample. The ROC_AUC of the AAindex to identify women with excess VAT accumulation was 0.80 (95% CI: 0.67–0.92) for VAT ≥100 cm^2^ and 0.72 (95% CI: 0.57–0.87) for VAT ≥130 cm^2^ (data not shown).

The sensitivity and specificity of the optimal glutamate threshold (≥34.6 μmol/L) and the use of the MetS or the HTW phenotype to identify individuals with excessive VAT are presented in Table 1. For VAT ≥100 cm^2^, the glutamate threshold had far better sensitivity than the other screening tools (83% for glutamate vs 52% for the MetS and 35% for the HTW), but the traditional risk algorithms had better specificity (71% for glutamate vs 83% for the MetS and 91% for the HTW). The glutamate threshold seemed to have better overall identification ability than the other screening tools for VAT ≥100 cm^2^, when targeting balance between sensitivity and specificity. The ability to identify participants with VAT ≥130 cm^2^ was less promising for all screening tools, but overall glutamate seemed to have the best equilibrium between sensitivity and specificity for this VAT area threshold.

**Table 1:** Sensitivity and specificity of different screening tools to identify women with excessive VAT accumulation

	VAT area ≥ 100 cm^2^		VAT area ≥ 130 cm^2^	
	Sensitivity	Specificity	Sensitivity	Specificity
Glutamate ≥34.6 μmol/L	83%	71%	79%	59%
MetS	52%	83%	57%	77%
HTW	35%	91%	36%	86%

*The optimal glutamate concentration threshold (≥34.6μmol/L) was determined with Youden’s Index calculations (J= sensitivity + specificity – 1). Sensitivity and specificity were defined as true positive/(true positive + false negative) and true negative/(true negative + false positive) respectively. MetS: metabolic syndrome, i.e. three or more of the following features: WC >88cm, TG ≥1.7mmol/L, HDL <1.3mmol/L, fasting glucose ≥5.6mmol/L and diastolic blood pressure (BP) ≥130mmHg or systolic BP ≥85mmHg; HTW: hypertriglyceridemic waist, i.e. WC ≥85cm and TG ≥1.5mmol/L*

### Variance analysis

**Fig. 2:**
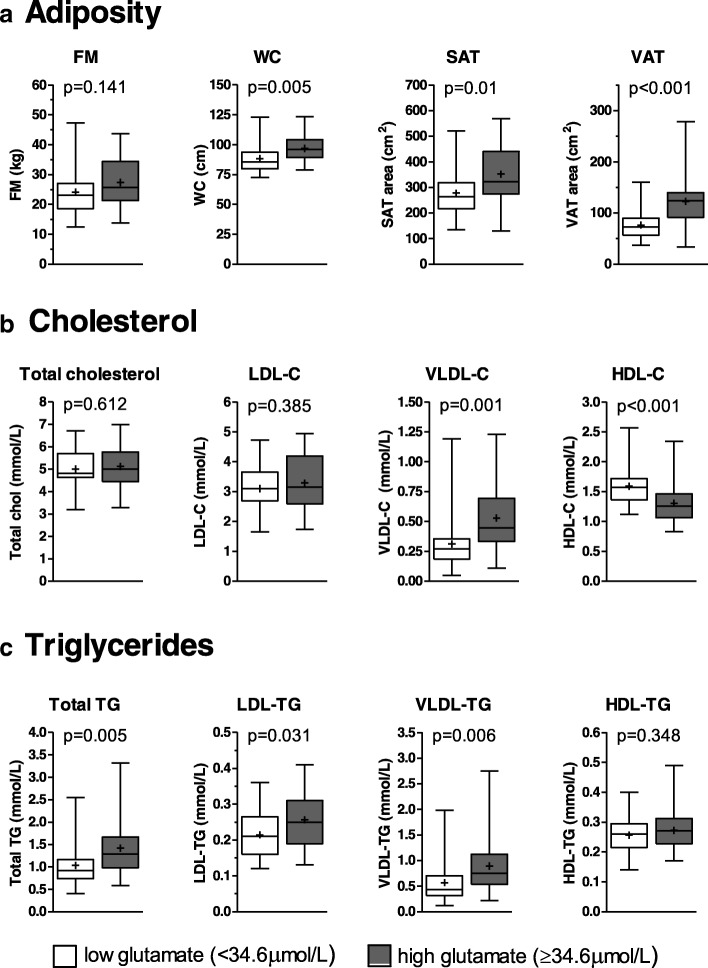
Comparison of adiposity, cholesterol and triglyceride values in women with low or high glutamate level. Results are presented as box-and-whisker plots; the box is the range between the lower (Q1) and upper (Q3) quartile, the horizontal line is the median (Q2), the cross sign (+) is the mean and the whiskers are the minimum and maximum. Women were considered as having a high glutamate level if it was ≥34.6 μmol/L (in grey) and low if it was less than 34.6 μmol/L (in white). *P*-values are from one-way ANOVA. *FM* fat mass, *WC* waist circumference, *SAT* subcutaneous adipose tissue, *VAT* visceral adipose tissue, *LDL* low-density lipoprotein, *VLDL* very-low-density lipoprotein, *HDL* high-density lipoprotein, *TG* triglycerides

Individuals were divided into low or high glutamate level subgroups according to the optimal glutamate threshold (≥34.6 μmol/L). Comparisons of adiposity indices as well as cholesterol and TG levels in plasma and lipoprotein fractions between the two subgroups are shown in Fig. 2.

Interestingly, fat mass (FM) was not significantly different between women with high or low glutamate, suggesting that concentrations of this analyte reflect body fat distribution rather than general adiposity. Furthermore, women with high circulating glutamate had, on average, a greater WC and abdominal SAT area. They also had greater VAT accumulation.

Total plasma cholesterol as well as cholesterol in the low-density lipoprotein (LDL) fraction was not significantly different between groups. Cholesterol in the very-low-density lipoprotein (VLDL) fraction was higher and that in the HDL faction was lower for the high glutamate level subgroup.

Total plasma TG as well as its levels in LDLs and VLDLs was higher in the high glutamate level subgroup, but was not significantly different in HDLs. Age, fasting glycaemia and insulinemia or HOMA-IR were not significantly different between individuals with high and low glutamate levels (data not shown). Glucose homeostasis variables were not significantly different in participants with versus those without the MetS or the HTW phenotype.

## Discussion

We aimed to determine the ability of glutamate concentration to identify individuals with visceral obesity and an altered metabolic profile. We showed that glutamate level was significantly associated with VAT and that it allowed identification of individuals with VAT area ≥100 cm^2^ and ≥130 cm^2^. To this end, the optimal glutamate threshold had a greater sensitivity, but a lower specificity than the MetS and the HTW phenotype. Furthermore, women with a high glutamate level had an altered metabolic profile, particularly regarding total TG levels and the amount of TG and cholesterol in their VLDLs. To our knowledge, this is the first study focusing on the potential of glutamate concentration as a biomarker of VAT accumulation and metabolic alterations.

Our results showed that glutamate is strongly correlated with VAT but less with SAT area (*r* = 0.46, *p* < 0.001 versus *r* = 0.33, *p* = 0.013). These results are consistent with those of the two other studies investigating the metabolomics of visceral obesity. Yamakado et al. reported that glutamate was strongly associated with VAT (*r* = 0.49, *p*-value not available) but weakly with SAT (*r* = 0.21, *p*-value not available) and Takashina et al. found that although glutamate was strongly correlated with VAT (*r* = 0.568, *p* < 0.001), it was not significantly associated with SAT (*r* = 0.196, *p* = 0.076).

Like us, Yamakado et al. found that glutamate had a good ability to identify individuals with VAT accumulation ≥100 cm^2^ (ROC_AUC: 0.75, 95% CI: 0.73–0.78). They created an index composed of 8 amino acids (valine, leucine, isoleucine, glutamate, glycine, alanine, tyrosine and tryptophan) which had a slightly higher VAT-predicting ability (ROC_AUC: 0.81, 95% CI: 0.78–0.83 for VAT ≥100 cm^2^). In our sample, this amino acid index showed virtually no improvement compared to the use of glutamate alone (ROC_AUC: 0.80, 95% CI: 0.67–0.92 for the index versus 0.78, 95% CI: 0.66–0.91 for glutamate alone, data not shown). The benefit of using a single metabolite as opposed to a composite score requires further analysis in other samples.

Takashina et al. investigated the association between amino acids and glucose homeostasis indices. They reported that glutamate was positively and significantly correlated with fasting glucose level (*r* = 0.439, *p* < 0.001), two hours glycaemia during an oral glucose tolerance test (OGTT, *r* = 0.302, *p* = 0.006) and the homeostasis-model assessment of insulin resistance (HOMA-IR) index (*r* = 0.292, *p* = 0.007). Other teams also have reported an association between glutamate and altered glucose metabolism, be it insulin resistance [15] or type 2 diabetes [16, 17]. Conversely, in our sample, glucose homeostasis was not different between women with high or low glutamate level. This discrepancy might be due to the fact that the insulin sensitivity range of our cohort was particularly narrow, which might have underestimated the association of glutamate levels with glucose homeostasis measurements.

Although the mechanism linking glutamate to VAT accumulation is not yet clear, we suggest that it may involve BCAA catabolism in visceral adipocytes. BCAAs can be metabolized in adipocyte mitochondria to generate substrates of the tricarboxylic acid (TCA) cycle and the first two steps of this pathway are common to all three BCAAs [18]. The first step is a transamination by the branched-chain-aminotransferase (BCAT) enzyme, in which α-ketoglutarate receives an amino group from the BCAA, producing glutamate and a branched-chain keto acid (BCKA). The subsequent step is a decarboxylation by the branched-chain-keto-acid dehydrogenase complex (BCKDC). Conversion of α-ketoglutarate to glutamate is an integral part of BCAA to BCKA transamination [19]. The fact that glutamate is a by-product of all 3 BCAAs catabolism may contribute to make it a stronger biomarker than individual BCAAs.

An increasing amount of evidence suggests gene expression down regulation of the two main BCAA catabolizing enzymes (BCAT and BCKDC) in adipocytes of obese individuals [6, 10, 20], which could partly explain the increased plasma BCAA and glutamate levels observed in obesity [21]. Herman et al. showed that obese mice BCAA catabolism was decreased in adipose tissue and not in skeletal muscle [22]. Furthermore, Nagao et al. demonstrated in an in vivo metabolic study that adipose tissue of obese mice (ob/ob as well as diet induced obese) produced significantly more glutamate than that of lean mice [23].

In humans, Lackey et al. compared adipose tissue gene expression of BCAT and BCKDC between metabolically impaired (presence of MetS) and healthy subjects. In SAT, no significant difference was observed. In VAT, metabolically impaired subjects had significantly lower BCKDC expression and a trend towards lower BCAT expression (*p* = 0.056) compared to healthy subjects [24]. Accordingly, the adipose tissue gene expression results in our sample showed that BCKDC was only decreased in VAT and not in SAT of the participants, whereas BCAT was decreased in both compartments [10]. These results suggest that although BCAA catabolism is decreased in both visceral and subcutaneous adipose tissue of obese subjects, BCKDC down regulation seems of particular importance in VAT. This could possibly explain the close association between glutamate level and VAT area that we and other teams have observed [8, 9]. According to this hypothesis, a BCKDC down regulation could block the metabolic pathway and cause glutamate accumulation. More studies are needed to confirm previous results and to assess whether other mechanisms are involved.

The main limitation of this study is that our sample is small and composed exclusively of lean-to-moderately obese women, making it difficult to extrapolate our results to other population. This is why we do not propose the optimal glutamate threshold found in this study to be used in the general population. More studies in larger and more diverse samples would be needed to establish such thresholds.

## Abbreviations

ANOVA: Analyses of variance
AUC: Area under the curve
BCAA: Branched-chain amino acid
BCAT: Branched-chain aminotransferase
BCKA: Branched-chain keto acid
BCKDC: Branched-chain keto acid deshydrogenase complex
BMI: Body mass index
BP: Blood pressure
CT: Computed tomography
DEXA: Dual-energy x-ray absorptiometry
FM: Fat mass
FSH: Follicle-stimulating hormone
HDL: High-density lipoprotein
HOMA-IR: Homeostasis-model assessment of insulin resistance
HTW: Hypertriglyceridemic waist
LDL: Low-density lipoprotein
MetS: Metabolic syndrome
MRI: Magnetic resonance imaging
OGTT: Oral glucose tolerance test
ROC: Receiving operator characteristic
SAT: Subcutaneous adipose tissue
TCA: Tricarboxylic acid
TG: Triglycerides
VAT: Visceral adipose tissue
VLDL: Very-low-density lipoprotein
WC: Waist circumference
